# Evaluation of Structural Changes and Molecular Mechanism Induced by High Hydrostatic Pressure in *Enterobacter sakazakii*

**DOI:** 10.3389/fnut.2021.739863

**Published:** 2021-09-23

**Authors:** Qiaoming Liao, Han Tao, Yali Li, Yi Xu, Hui-Li Wang

**Affiliations:** ^1^Engineering Research Center of Bio-process, Ministry of Education, Hefei University of Technology, Hefei, China; ^2^School of Food Science and Engineering, Hefei University of Technology, Hefei, China

**Keywords:** *E. sakazakii*, high hydrostatic pressure, biofilm, flagellar proteins, mechanism

## Abstract

The contamination of infant milk and powder with *Enterobacter sakazakii* poses a risk to human health and frequently caused recalls of affected products. This study aims to explore the inactivation mechanism of *E. sakazakii* induced by high hydrostatic pressure (HHP), which, unlike conventional heat treatment, is a nonthermal technique for pasteurization and sterilization of dairy food without deleterious effects. The mortality of *E. sakazakii* under minimum reaction conditions (50 MPa) was 1.42%, which was increased to 33.12% under significant reaction conditions (400 MPa). Scanning electron microscopy (SEM) and fluorescent staining results showed that 400 MPa led to a loss of physical integrity of cell membranes as manifested by more intracellular leakage of nucleic acid, intracellular protein and K^+^. Real-time quantitative PCR (RT-qPCR) analysis presents a downregulation of three functional genes (*glpK, pbpC*, and *ompR*), which were involved in cell membrane formation, indicating a lower level of glycerol utilization, outer membrane protein assembly, and environmental tolerance. In addition, the exposure of *E. sakazakii* to HHP modified oxidative stress, as reflected by the high activity of catalase and super oxide dismutase. The HHP treatment lowered down the gene expression of flagellar proteins (*fliC, flgI, fliH*, and *flgK*) and inhibited biofilm formation. These results determined the association of genotype to phenotype in *E. sakazakii* induced by HHP, which was used for the control of food-borne pathogens.

## Introduction

*Enterobacter sakazakii* is a motile, non-spore formation and rod-shaped Gram-negative organism. It is an emerging food-borne pathogen that is found in several foods, such as skim milk, meat, chicken skin, fruits, and cheese (1). *E. sakazakii* can form biofilm, which is a spatially organized dynamic ecosystem, against environmental stress and host immune surveillance mechanisms (2). It can attach to abiotic materials such as silicon, latex, polycarbonate, stainless steel, glass, and polyvinyl chloride, which results in a series of infectious diseases such as neonatal sepsis, meningitis, and brain abscess (3). *E. sakazakii* can survive in a wide range of temperatures from 4 to 47°C, exhibiting substantial resistance to heating process. Edelson-Mammel et al. (4) isolated 12 *Enterobacter* strains from rehydrated powdered infant formula that could survive at 58°C. Perez et al. (5) studied the effectiveness of pulsed electric field treatment on the inactivation of *E. sakazakii*, and they found that higher field strength and longer treatment time increased the inactivation. Lee et al. (6) showed that there was no regrowth of *E. sakazakii* in contaminated infant powder after gamma-irradiation at 5 kGy. More and more researchers are focusing on green and efficient non-thermal sterilization instead of traditional heating process to keep the sensory, nutritional, and functional ingredients of food.

High hydrostatic pressure has been demonstrated to be an effective non-thermal processing method for the pasteurization and sterilization of dairy food (7). The pressure is applied on the interior and surface of foods through liquid instead of traditional thermal treatment. The potential application of HHP to *E. sakazakii* inactivation is focused on the process of wet mixture treatment before spray drying and the production of a novel non-thermally treated pasteurized liquid infant milk formula. The first study on *E. sakazakii* inactivation by HPP was conducted by Gonzalez et al. (8) using a reference medium. Inactivation levels between 2 and 6 log10 cycles were achieved. Arroyo et al. (9) reported that HHP resulted in sublethal injuries to the outer and cytoplasmic membranes in *E. sakazakii*. Pressure-holding time, pressure level, and food matrix also affect the inactivation of *E. sakazakii* (10). Sublethal injuries to the outer and cytoplasmic membranes were detected after HHP. Scanning electron micrographs indicated that cellular envelope and intracellular damages of *E. sakazakii* cells were apparent after 300 and 400 MPa for 5 min compared with the untreated cells, and a progressive increase of injured cells with increased pressure treatment was observed. *E. sakazakii* was sensitive to high pressure processing treatment, and high-pressure processing treatment with 400 MPa for 3 min can be used to control *E. sakazakii* contamination in milk samples (11). This article aims to determine the relationship between HHP-induced structural changes and molecular variations that occur in *E. sakazakii* by plate counting, fluorescence staining, SEM, cellular leakage, oxidative enzyme activity, and RT-qPCR analyses. Applying new findings in Cronobacter spp. sterilization techniques would be of great help in assuring product quality.

## Materials and Methods

### Bacterial Strain and Incubation Conditions

Enterobacter *sakazakii* (ATCC 29544) was purchased from BeNa Culture Collection (Beijing, China). Strain activation was performed by following the instructions of the supplier. First, the activated strains stored at −20°C were transferred into a test tube containing 5-ml nutrient broth (Sinopharm Chemical Reagent Co., Ltd., Beijing, China) at 37°C in a constant temperature incubator under 200-rpm shaking speed conditions overnight. Then, 100 μl of the incubated strains were transferred in a fresh nutrient broth (5 ml) and cultured under same conditions for approximately 4 to 5 h until the concentration of cells reached 10^8^ colony-forming unit (CFU)/ml.

### HHP Treatment

The bacteria were cultured in a nutrient broth medium for 6 h and subjected to centrifugation at 5,700 × g for 3 min. The cell precipitates were washed twice with phosphate-buffered saline (PBS) and then centrifuged by following the above procedure. The pellets were resuspended in PBS (1 ml) to a cell destiny of 10^8^ CFU/ml. The HHP experimental method used in this study is described in Yamin et al. (12). The cell suspension (2 ml) was heat-sealed in sterile polyethylene (Wangshi Packaging, Hebei province, China) after exclusion of the air bubbles and subjected to 0-, 50-, 100-, 200-, 300-, and 400-MPa treatments at 25°C for 10 min, with 3-min pressurization/30-s depressurization. The HHP treatment was administered using a 3-L-capacity pressure vessel (Bao tou KeFa High Pressure Technology Co., Ltd, Bao tou, China), with water as the transmission fluid.

### Plate Counting Method

Viable colonies were enumerated by plate dilution colony counting. All the treated groups of samples were serially diluted with a 0.85% NaCl solution in a ratio of 1:9. Subsequently, the aliquot (50 μl) of the cultures in solid nutrient broth was plated and incubated at 37°C for 24 h. Each experiment was performed in triplicate.

### Intracellular Leakage Measurement

Aliquots of 3 ml of cell cultures exposed to different intensities of pressure were centrifuged at 4,000 × g for 5 min. The supernatant containing nucleic acid was gradually subjected to filtration using a 25-mm and 0.22-μm-diameter filter. Total nucleic acid content was quantified with a micro plate spectrophotometer (Infinite 200 PRO; Tecan, Mannedorf, Switzerland) with absorbance at 260 nm.

The leakage of protein was measured by bicinchoninic acid (BCA) protein method with a protein assay kit (A045-4-2, Nanjing Jiancheng Bioengineering Institute, Jiangsu, China). One milliliter of the cell sample was mixed with 9 ml of PBS solution (0.1 mol/L, pH 7.4) and then centrifuged at 3,500 × g for 10 min, and 10 μl of the supernatant was incubated with BCA buffer (250 μl) at 37°C for 30 min before being subjected to measurement using a microplate spectrophotometer (Infinite 200 PRO; Tecan, Mannedorf, Switzerland) with maximum absorption at 562 nm.

The leakage of K^+^was measured by following the instructions in the K^+^assay kit (C001-2-1, Nanjing Jiancheng Bioengineering Institute, Jiangsu, China). The bacteria cell culture was mixed with deionized water in a ratio of 1:9 and centrifuged at 2,500 × g for 10 min, and then 20 μl of the supernatant was precipitated with 180 μl of protein deposits and centrifuged at 3,500 × g for 5 min. Fifty picoliters of the supernatant was incubated with an Na-TPB buffer (200 μl) for 5 min before being subjected to measurement using a microplate spectrophotometer (Infinite 200 PRO, Tecan, Mannedorf, Switzerland) at 450 nm.

### Measurement of Cellular Enzyme Activity

Superoxide dismutase enzyme activity was estimated using a superoxide dismutase (SOD) assay kit (A001-3-1, Nanjing Jiancheng Bioengineering Institute, Jiangsu, China). All the *E. sakazakii* samples were centrifuged at 1,000 × g for 10 min and the sediments were cultured with 1 ml of PBS (pH = 7) to get homogenized for 3 min; then, 20 μl of the culture was mixed with 20 μl of the enzyme solution and 200 μl of the substrate solution at 37°C for 30 min before being subjected to measurement using a microplate spectrophotometer (Infinite 200 PRO; Tecan, Mannedorf, Switzerland) with 450 nm absorbance.


$$\begin{array}{l} \text{SOD inhibition ratio }(\%)=[({\text{A}}_{\text{C}}\;-{\text{ A}}_{\text{BC}})\;-\;({\text{A}}_{\text{E}}\;-\;{\text{A}}_{\text{BE}})]/({\text{A}}_{\text{C}}\\ -\;{\text{A}}_{\text{BC}})\;\times \;100\%\\ \text{SOD activity }(\text{U}/\text{mg prot})\\ =\text{ SOD inhibition ratio}\times /\text{protein concentration }(\text{mg prot}/\text{ml}) \end{array}$$

Catalase (CAT) activity was measured by following the instructions in the CAT assay kit (A007-1-1, Nanjing Jiancheng Bioengineering Institute, Jiangsu, China). The cell culture was prepared as SOD procedure: All the *E. sakazakii* samples were centrifuged at 1,000 × g for 10min and the sediments were cultured with 1 ml of PBS (pH = 7) to get homogenized for 3 min which has been added. Then, 1 ml of NO 0.3 solvent and 0.1 ml of NO 0.4 solvent were immediately added before measurement using a microplate spectrophotometer (Infinite 200 PRO; Tecan, Mannedorf, Switzerland) at 405 nm absorbance.


$$\begin{array}{l} \text{CATactivity}(\text{U}/\text{mg prot})\\ \;=\;({\text{OD}}_{\text{blank}}\;-\;{\text{OD}}_{\text{sample}})\;\times \;271/(60\\ \;\times \text{ samplecapacity})\text{/proteinconcentration}(\text{mg prot}/\text{ml}): \end{array}$$

A_C_: the absorbance of control group at 450 nm
A_BC_: the absorbance of blank control group at 450 nm
A_E_: the absorbance of experimental group at 450 nm
A_BE_: the absorbance of experimental blank group at 450 nm.

Protein concentration was measured by following the instructions in the protein assay kit (A045-4-2, Nanjing Jiancheng Bioengineering Institute, Jiangsu, China).

### SEM Analysis

The samples were centrifuged (5,700 × g) for 3 min. The sediment was mixed with 2.5% glutaraldehyde overnight at 4°C to fix. Then, the fixed cells were washed with phosphate buffer and dehydrated in graded ethanol solutions (50, 70, 95, and 100%), stuck to the conductive adhesive tape, coated with gold-palladium, and observed with a scanning electron microscope (X650; Hitachi, Tokyo, Japan).

### Fluorescent Staining Procedure

Fluorescent dye propidium iodide (PI) was used to evaluate cell permeabilization. The HHP-treated *E. sakazakii* (50 and 400 MPa) suspensions in phosphate buffered saline (PBS) were mixed with 50 μl of the PI solution (5 mg/ml). The mixture was placed in the dark at 25°C for 10 min and then centrifuged (5,700 × *g*) for 3 min at 4°C. The precipitate was washed with PBS twice until no excess dye was left. Fluorescence was measured with a fluorescence microscope (ECLIPSE Ti2; Nikon, Tokyo, Japan).

### Quantification of Biofilm Formation

Biofilm quantification was performed by crystal violet (CV) assay. Biofilms formed in 96-well polystyrene microtiter plates after the HHP treatment. Non-adherent cells were washed softly with phosphate-buffered saline (PBS) after 12, 24, and 48 h of growth and fixed under 60°C for 1 h. The biofilms were stained with 200 μl of 0.1% crystal violet (CV) for 20 min and washed with PBS (pH = 7) three times until no CV was left. First, the biofilms were imaged using the fluorescence microscope (ECLIPSE Ti2; Nikon, Tokyo, Japan) and then dissolved in 33% acetic acid. The OD values of each well were determined at 590 nm using the microplate spectrophotometer (Infinite 200 PRO; Tecan, Mannedorf, Switzerland).

### RNA Isolation and Real-Time Quantitative Polymerase Chain Reaction (RT-qPCR) Assay

The cells were pelleted by centrifugation at 5,000 rpm/min for 1 min, washed three times, and resuspended in PBS (pH = 7). Total RNAs were extracted from *E. sakazakii* using RNeasy Protect Bacteria Mini Kit (QIAGEN, Shanghai, China) after the HHP treatment. The quality, integrity, and concentration of the RNA were measured. Subsequently, the RNA was reverse-transcribed into cDNA with First-Stand cDNA Synthesis SuperMix (Transgen Biotech, Beijing, China) and stored at −20°C, resulting in the first stands of total cDNA. The primers custom-synthesized by General Biosystems LTD (Anhui, China) are listed in Table 1. Amplification and detection were carried out using LightCycle96 Q-PCR (Roche, Basel, Switzerland). The relative expression of each gene was quantified using 16S rRNA as internal control. All the samples were analyzed in triplicate.

**Table 1:** Primers used for real-time quantitative PCR (RT-qPCR) analyses.

**Primers**	**Sequences (5^′^-3^′^)**	**Gene**
GlpKF	TGGCGAACAACTTCCTGA	*GlpK*
GlpKR	CGCTCGGTGGTTTCAATAC	
PbpCF	TTCGCAACTTGGCTATTCC	*PbpC*
PbpCR	AAGCGTAGTGGTGATTTTGG	
OmpRF	TGGTGCTTGATCTGATGCTTC	*OmpR*
OmpRR	AGGGTTGAACGGTTTAGGAAT	
FliCF	CTTACAGCGTATCCGTGAGC	*FliC*
FliCR	GCCGTTGAAGTTAGCACCA	
FliHF	ACCTGATTAAGCAGATCCAGAC	*FliH*
FliHR	TTCATCGGCGGAGACTTTG	
FlgKF	CGTTCTGGGGCAGTCTAACA	*FlgK*
FlgKR	GACCATATCGTCGATTTTCG	
FlgIF	GATTCCTGCTGTTGCTCGTC	*FlgI*
FlgIR	TCAGGCTCTGGGTGGTAAA	

### Statistical Analysis

All the experiments were carried out in triplicate. Statistical analysis was performed using the SPSS software (version 19.0; IBM; Armonk, NY, United States). An analysis of variance was performed on the results by an independent sample test, and the data were presented as the mean values ± SD. A *P*-value lower than 0.05 indicated a significant difference.

## Results and Discussion

### Inactivation of *Enterobacter sakazakii* With HPP

**Figure 1:**
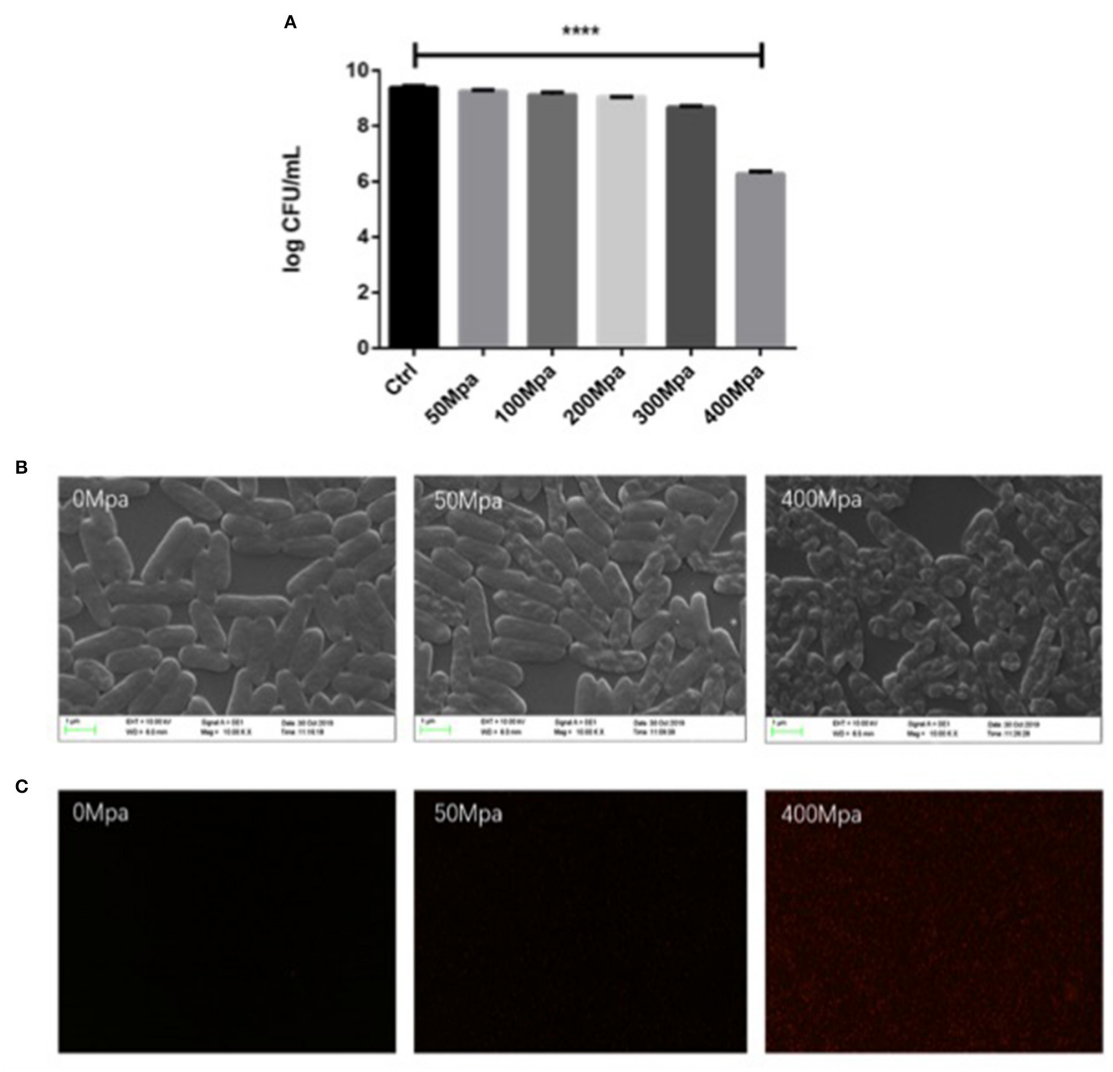
**(A)** Survival curves of *Enterobacter Sakazakii* in phosphate buffer with HHP treatment. **(B)** Scanning electron microscopy (SEM) micrographs and **(C)** uptake of propidium iodide by *E. sakazakii* cells. Asterisks indicate difference between control and different high hydrostatic pressures (HHPs) at ****P < 0.0001.

The extent of the inactivation of *E. sakazakii* exposed to the pressure treatment was assessed by plate count analysis (Figure 1A). This result revealed that the percentage of survival of the cells decreased with increase in pressure. The mean initial count of *E. sakazakii* in PBS was 9.39 log CFU/ml, which was slightly reduced by the 50-MPa treatment by 0.35 log but was significantly (*P* < 0.001) decreased to 3.11 log CFU/ml under 400 MPa conditions. This showed that the organism tolerated the pressure with little effect on viability when 50 MPa was reached. Similar results were reported by Gonzalez et al. (8) and Perez et al. (5). Inactivation levels between 2 and 6 log10 cycles were achieved. Thus, 50 and 400 MPa were used as the minimum treatment limit and significant treatment conditions for subsequent structural and molecular analyses.

Structural changes in *E. sakazakii* were observed by propidium iodide (PI) uptake and scanning electron microscopy (SEM). PI analysis exhibited higher permeability of HHP-treated bacterial cell membrane, since the fluorescent agent could penetrate into the internal structure of the damaged cell membrane (13). As shown in Figure 1C, there is no detection of PI intracellular accumulation observed in both the control group and the 50-MPa-treated group, indicating live bacteria with integrated cell membrane. In the case of 400 MPa, most of the bacteria were stained red, implying that HHP led to incomplete outer and inner membranes, which is in agreement with the results of the plate counting method. Higher HHP treatment resulted in more damage in cell.

The survival changed with modified morphology, which is present in Figure 1B. The bacteria cell kept intact and the structure of cell was full under 50 MPa conditions. However, the original structure of *E. sakazakii* cells treated with 400 MPa was seriously damaged after inactivation, and it presented more pimples and swellings on the surface, and had an enlargement of electron-transparent regions in the bacterial cytoplasm. It indicated a damaged outer membrane of HHP-treated *E. sakazakii*, which was consistent with the results of Lee et al. (14), who found that HHP caused disruption of bacterial cell membrane and cell wall.

### Effect of HHP on Intracellular Leakage

**Figure 2:**
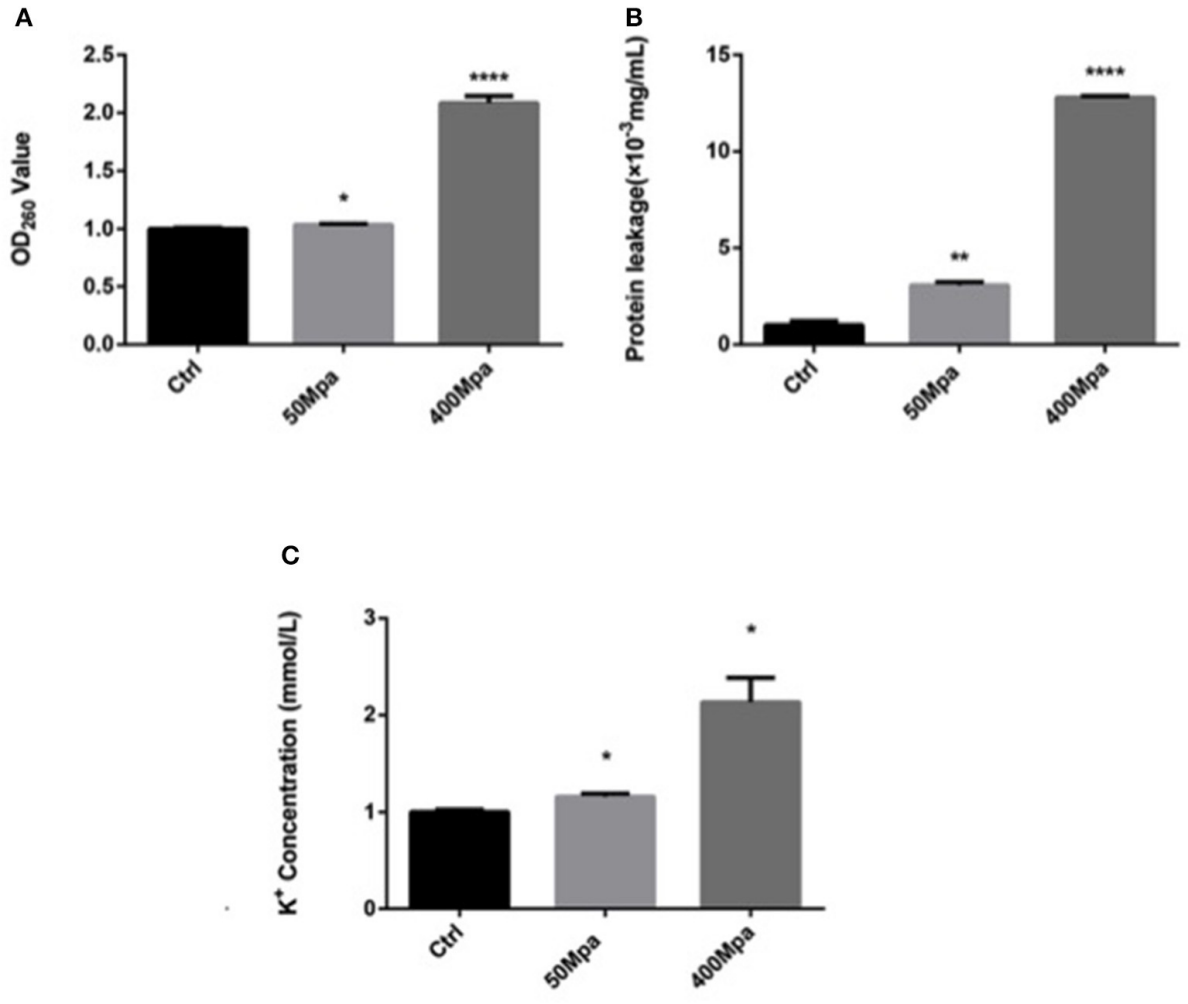
Intracellular leakage after HHP treatment. **(A)** Nucleic acid leakage, **(B)** protein leakage, **(C)** potassium leakage. Asterisks indicate difference between control and different HHPs at *P < 0.05, **P < 0.01, ***P < 0.001, and ****P < 0.0001.

Figure 2 shows the leakage of DNA, intracellular protein, and K^+^ of *E. sakazakii* affected by the 50- and 400-MPa treatments. The 400-MPa treatment induced an obvious increase in intracellular protein release from 3.19 to 13.55 μg/ml, while no significant changes were noted in the 50-MPa group. A similar trend was also observed in the content of DNA and K^+^. Nucleic acid and protein are important in bacterial cell growth, while K^+^ affects cell internal balance (15). These intracellular leakages can be used as makers of cell membrane disruption. When the microorganism was exposed to severe environmental stress, the microbial cell membrane was destroyed, leading to different extents of loss of intercellular contents from the interior of the cell, cell lysis, and, finally, cell death.

### Effect of HHP on Key Cell Membrane Genes

**Figure 3:**
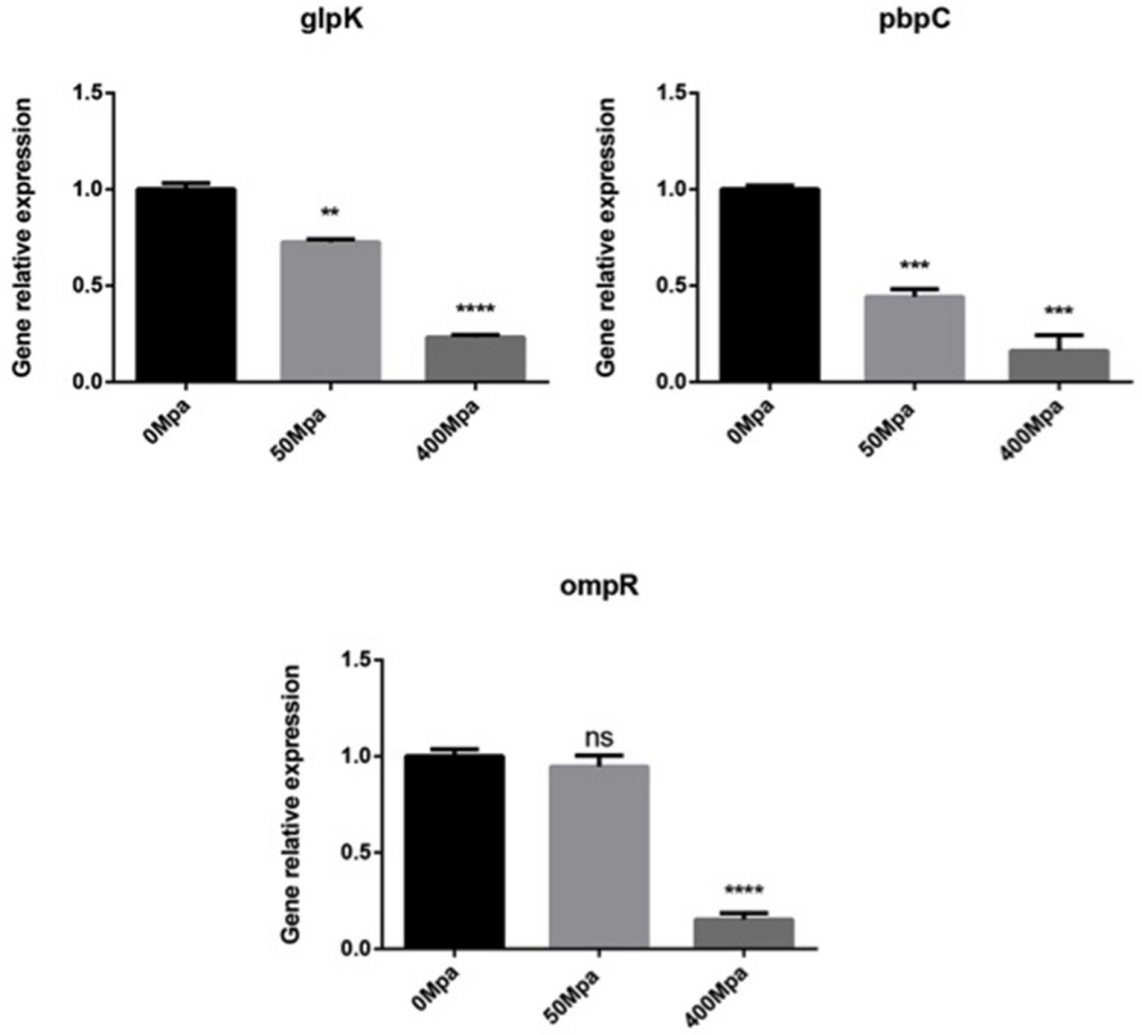
Expression of selected cell membrane genes in *E. sakazakii* treated with HHP. Asterisks indicate difference between control and different HHPs at *P < 0.05, **P < 0.01, ***P < 0.001, and ****P < 0.0001.

In order to understand the association of genotype to phenotype, three functional genes (*glpK, pbpC*, and *ompR*) that correspond to the progress of cell membrane formation were quantified by qPCR (Figure 3). The RT-PCR analysis revealed that the transcript level of the membrane genes was downregulated by the HHP treatment in a significant way (*P* < 0.05. Glycerol kinase (*glpK*) could catalyze the conversion of glycerol to gly-cerol-3-phosphate, the first step in glycerol catabolism, while *pbpC* could contribute to the biogenesis of surface organelles by interaction with a patch of the cytoskeletal protein bactofilin. HHP reduced *glpK* and *pbpC* activity, indicating lower glycerol utilization rate and outer membrane protein assembly, which was disadvantageous for cell growth. *ompR* is a response regulator in protecting cells against environmental stress through phosphorylation to elicit an adaptive response. The HHP treatment induced less *ompR* transcript in the *E. sakazakii* strain where it failed to repair damage caused by osmotic stress. This was consistent with the results observed in Gram-negative bacteria where the regulatory system of stress responses was triggered against limited conditions (16).

### Effect of HHP on Anti-oxidative Enzymes Activity

**Figure 4:**
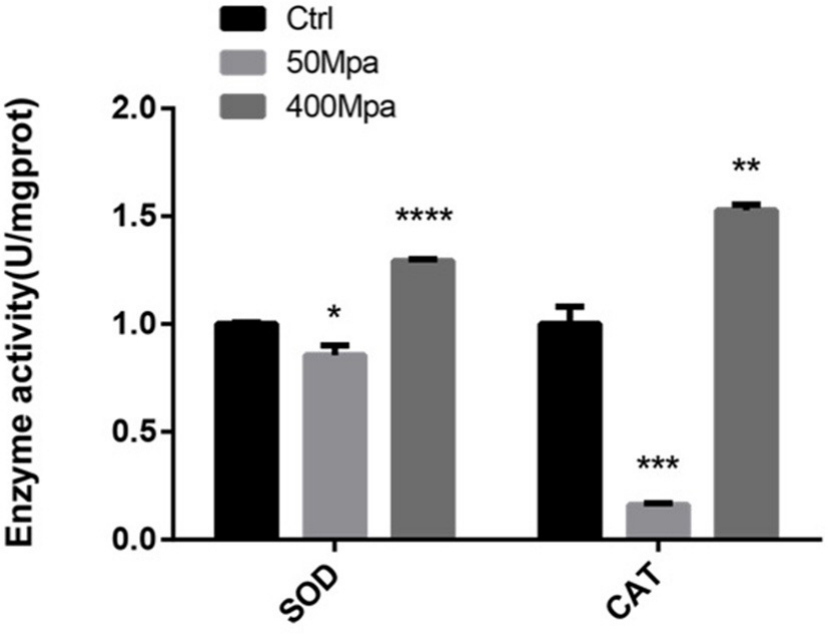
Enzyme activity of superoxide dismutase (SOD) and catalase (CAT) of *E. sakazakii* with different pressures. Asterisks indicate difference between control and different HHPs at *P < 0.05, **P < 0.01, ***P < 0.001, and ****P < 0.0001.

The inhibitory action of HHP is mainly regarded as the generation of ROS in the bacterial cell. The major regulation of antioxidant levels contains superoxide dismutase (SOD) and catalase (CAT), or non-enzymatic antioxidants (17). Figure 4 shows the bacterial defense mechanisms against oxidative stress by HHP. The activity of SOD and CAT decreased at 50 MPa from 83.61 and 120.27 U/mg prot to 71.54 and 19.41 U/mg prot, respectively. This suggested that the intracellular redox homostasis in *E. sakazakii* was broken by the HHP treatment (18). However, increasing the pressure showed an opposite trend. The 400-MPa treatment increased the activity of SOD and CAT to 108.72 and 183.77 U/mg prot, respectively. The initial inhibition of the SOD and CAT activity in the preliminary 50-MPa HHP treatment might be due to the limited conditions to make stress response for *E. sakazakii*. The bacteria had the ability to adapt and tolerate the lower pressure stimuli. When high pressure was applied, the enzyme activity was increased for bacterial oxidative stress response to external stimuli, which caused damage to the cells (19). Other studies have been conducted to analyze the endogenous intracellular oxidative stress in *Escherichia coli* and *Mytilus galloprovincialis*, which have shown an increase in the SOD and CAT activity when exposed to HHP (20, 21).

### Effect of HHP on Biofilm Formation System

**Figure 5:**
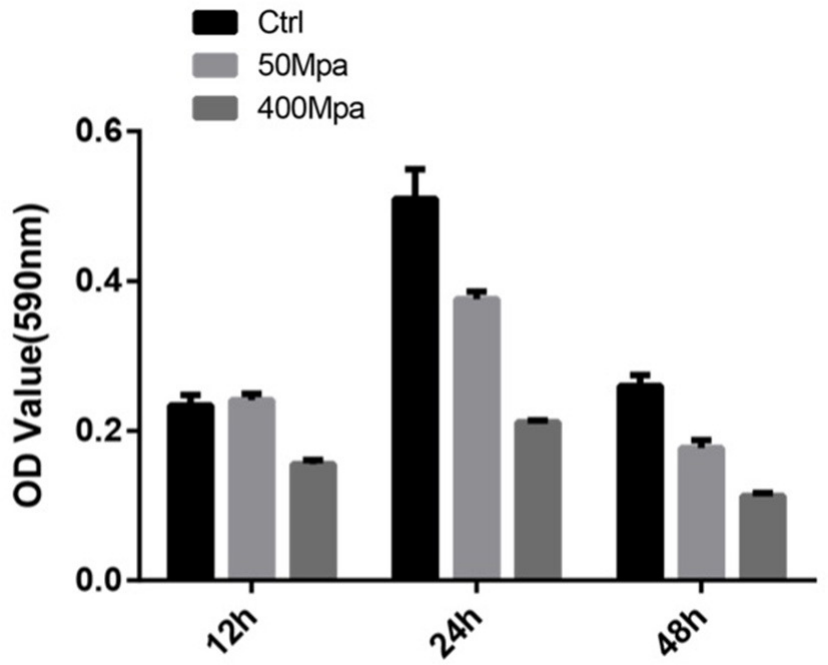
Biofilm formation of *E. sakazakii* incubated at 12, 24, and 48 h. Vertical error bars represent standard deviations of three replicate samples.

Figure 5 show the biofilm-producing ability of *E. sakazakii* incubated at 12, 24, and 48 h. After 24-h incubation, biofilm formation was increased while an opposite trend was observed at 48 h. This could be attributed to the biofilm dispersion induced by starvation (22). When HHP was applied, the biofilm-producing ability was reduced. The quantity of biofilm was significantly decreased by 45% in the presence of 400 MPa after 24-h incubation. This suggests that HHP could inhibit *E. sakazakii* cells from adhering to solid surfaces, which is in agreement with the cell membrane variations mentioned in sections of Intracellular Leakage. The biofilm-producing ability of *E. sakazakii* strains under different environmental stress conditions has been described previously in a number of studies (23).

**Figure 6:**
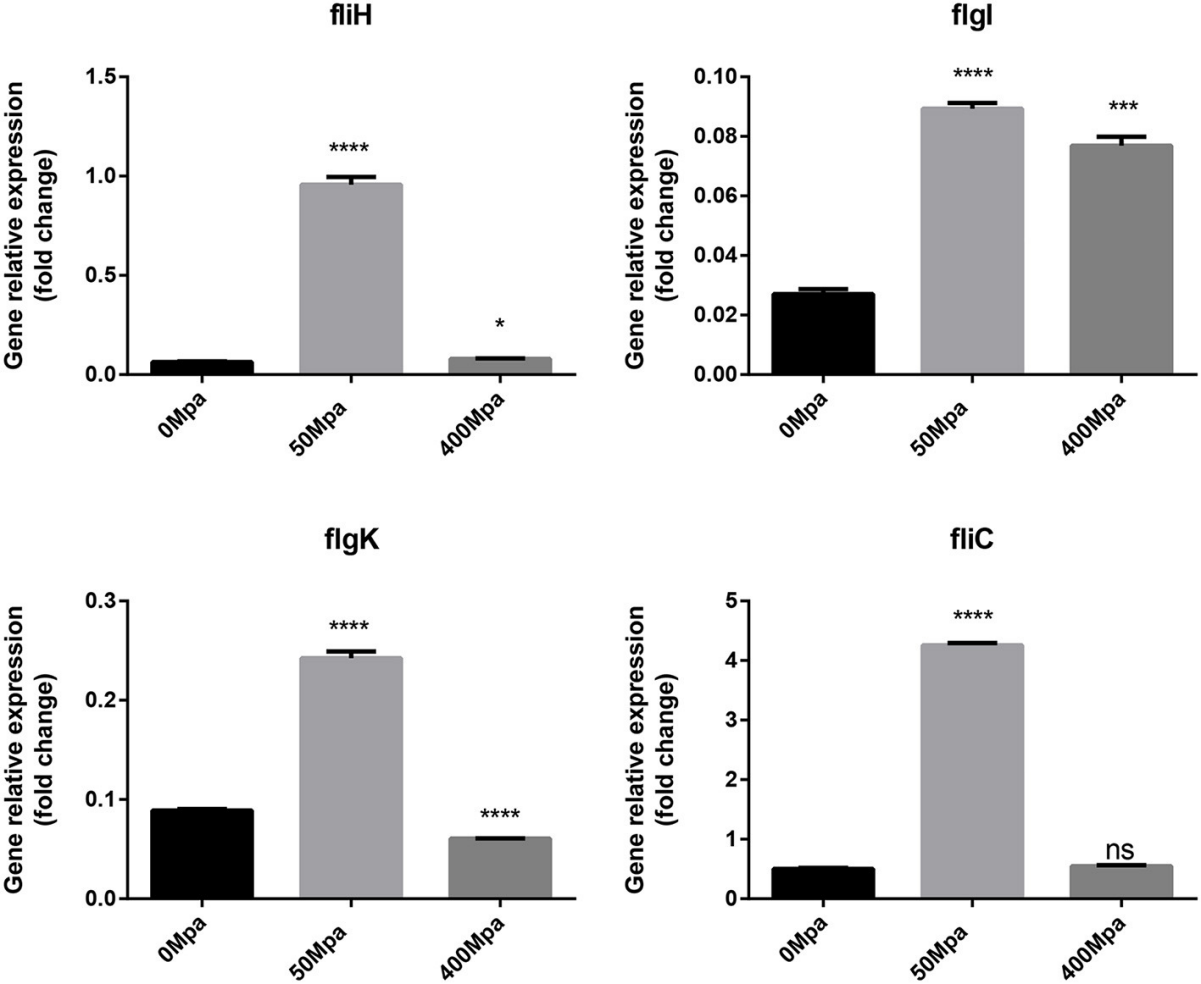
Expression of flagellin genes in *E. sakazakii* treated with HHP. Asterisks indicate difference between control and different HHPs at *P < 0.05, **P < 0.01, ***P < 0.001, and ****P < 0.0001.

The changed biofilm formation was controlled by their related virulence genes (*fliH, fliC, flgI*, and *flgK*). As shown in Figure 6, it is found that the flagellin genes are increased at 50 MPa and decreased at 400 MPa. *FliC* and *FliD*, two flagellar cap proteins, were present on the F1 gene locus of the flagellar regulon, while *flgK* and *flgI* coded for a hook-associated protein that stabilized the hook-filament junction together with *FlgL* (24). They were involved in the growth and diffusion of biofilm as a function of adhesion and colonization. The mRNA expression of biofilm-related genes slightly increased after the low-pressure treatment compared with the control, which could have contributed to bacterial chemotaxis. The flagellum synthesis in *E. sakazakii* was activated against poor environment for protection. However, the high-pressure conditions damaged the protection mechanism and resulted in loss of the ability to self-regulate.

## Conclusion

In summary, HHP influenced cell membrane-related cell behaviors, such as metabolism utilization, outer membrane protein assembly, and stress responses in *E. sakazakii*. The variations in genotypes were reflected as lower level of cell membrane permeability, higher leakage of intracellular substances, and cellular deformations. Meanwhile, HHP increased bacterial oxidative stress response and disrupted biofilm formation by inhibiting cell adherence and extracellular matrix production. These findings will provide an understanding of the associations between bacterial genotype and phenotype induced by HHP.

## Data Availability Statement

The raw data supporting the conclusions of this article will be made available by the authors, without undue reservation.

## Author Contributions

QL: methodology and formal analysis. HT: investigation and writing—original draft preparation. YX: writing—review and editing. YL: visualization. H-LW: supervision and funding acquisition. All authors have read and agreed to the published version of the manuscript.

## Funding

This study was supported by the National Key Basic Research Program of China (Grant Nos: 2018YFC1602201 and 2018YFC1602204) and the Fundamental Research Funds for the Central Universities (Grant No: JZ2020HGTB0043).

## Conflict of Interest

The authors declare that the research was conducted in the absence of any commercial or financial relationships that could be construed as a potential conflict of interest.

## Publisher's Note

All claims expressed in this article are solely those of the authors and do not necessarily represent those of their affiliated organizations, or those of the publisher, the editors and the reviewers. Any product that may be evaluated in this article, or claim that may be made by its manufacturer, is not guaranteed or endorsed by the publisher.
